# Genome-Wide Identification of Alternative Splicing in *Botrytis cinerea* During Infection Stage of *Solanum lycopersicum*

**DOI:** 10.3390/microorganisms13020360

**Published:** 2025-02-07

**Authors:** Ping Lu, Yun Wang, Xiaoli Wang, Jiaqi Wang, Dan Wang, Haojie Shi

**Affiliations:** 1 College of Advanced Agricultural Sciences, Zhejiang A&F University, Hangzhou 311300, China; luping@zafu.edu.cn (P.L.); wangyun@stu.zafu.edu.cn (Y.W.); wjq20001002@163.com (J.W.); 2 Jiangsu Provincial Key Construction Laboratory of Probiotics Preparation, Huaiyin Institute of Technology, Huaian 223003, China; xlwang@hyit.edu.cn; 3 State Key Laboratory of Subtropical Silviculture, School of Forestry and Biotechnology, Zhejiang A & F University, Hangzhou 311300, China

**Keywords:** *Botrytis cinerea*, RNA-Seq, infection, alternative splicing

## Abstract

Alternative splicing plays a crucial role in enhancing the protein diversity of eukaryotic genomes. However, alternative splicing has not been extensively studied in *Botrytis cinerea*. In this study, we examined the distribution and regulation of alternative splicing in the filamentous plant pathogenic fungus *B. cinerea* through strand-specific RNA sequencing at various stages of infection of *Solanum lycopersicum*. During infection (pre-penetration and biotrophic stage), most spliceosome genes had upregulated expression levels, indicating that splicing is altered at this stage. A total of 3308 genes underwent alternative splicing in *B. cinerea*, resulting in 7466 alternative splicing events, most of which were stage-specific. Transcripts generated through alternative splicing typically exhibit lower expression levels, coding potential, and functional domains, which are more prevalent during the hyphal phase compared to the infestation phase. To conclude, our research offers an extensive analysis of the genome-wide alternative splicing in *B. cinerea* throughout the infection process, acting as a significant resource for further clarifying the pathogenic mechanisms associated with *B. cinerea*.

## 1. Introduction

Splicing the 5′ and 3′ end processing of precursor messenger RNA is an essential step in mRNA maturation. However, the processing of pre-mRNA is a highly regulated and dynamic process. This dynamic process arises from alternative transcription initiation (ATI), alternative splicing (AS), and alternative polyadenylation (APA). The transcriptional diversity generated by these events greatly increases the complexity of transcript structures, resulting in a wide variety of protein products [1]. Among them, alternative splicing is a ubiquitous regulatory mechanism that affects the coding region of the gene. Most pre-mRNAs can be spliced in an invariant manner, resulting in different isoforms from one gene and greatly increasing the genome diversity, and this process is called alternative splicing, which is significant for the regulation of cell, tissue and condition adaption [2,3]. Alternative splicing is a global post-transcriptional control mechanism that plays an important role in a variety of biological processes. In humans, neuron-specific transcript isoforms generated by alternative splicing play important roles in neurogenesis and contribute to neuronal multifunctionality by helping to orchestrate specialization processes [4,5]. The OsMADS1, an alternatively spliced transcript from a natural variation, leads to increased expression of MST4, thereby increasing the granule thickness and improving granule quality in rice [6].

*Saccharomyces cerevisiae* has been utilized as a model fungus to investigate the mechanisms of pre-mRNA splicing. However, only a limited number of examples of alternative splice site usage have been documented in this organism, which contains approximately 300 introns [7,8]. With the rapid development of omics technology, the study of alternative splicing is gradually increasing in plant pathogenic fungi. Through third-generation sequencing technology, it was found that approximately 42.7% of genes in *Fusarium graminearum* undergo alternative splicing [9]. In *Magnaporthe oryzae*, approximately 2413 genes are alternatively spliced, of which 499 genes can be regulated by infection-specific alternative splicing [10]. Approximately 50% of the multi-exon genes in the *Verticillium dahliae* genome are regulated by alternative splicing, including 4033 intron retention, 582 alternative 3′-Splice Site (SS), and 379 alternative 5′ Splice Site (SS) [11]. In contrast to humans and animals, alternative splicing (AS) is not well studied in plant pathogenic fungi, such as *B. cinerea*, which has a wide host range.

*B. cinerea*, the causative agent of gray mold, can infect more than 1400 species of plants due to its excellent reproductive capacity and multi-faceted infection strategies [12]. Controlling this fungal pathogen poses significant challenges due to its extensive host range and capacity to persist in the environment. Currently, the treatment of gray mold predominantly relies on chemical methods [13]. An in-depth understanding of the transcriptional regulatory mechanisms during the infection stage of plant pathogenic fungi is the basis for finding potential control strategies.

A comprehensive understanding of the transcriptional regulatory mechanisms involved during the infection stage of plant pathogenic fungi serves as a foundation for identifying potential control strategies. During the infection of the host by *Sclerotinia sclerotiorum*, 641 genes exhibiting alternative splicing were differentially expressed, including 16 putative transcripts that produced signal peptides [14]. In *M. oryzae*, approximately 2413 genes exhibited alternative splicing, with 32.7% of these events occurring specifically during the infection, particularly after initial hyphal penetration into the host cell [10]. Recent research indicates that the expression levels of the same alternative splicing event in a specific plant pathogenic fungus are dynamically adjusted in response to the infection of various host plants [15]. Some studies have shown that novel isoforms produced through alternative splicing play a crucial role in the growth and development of plant pathogenic fungi, as well as their capacity to infect host organisms. In *F. graminearum*, there were two transcript isoforms of Fgsrp1 (a serine/arginine-rich protein) resulting from alternative splicing, and both isoforms were essential for its normal growth and deoxynivalenol (DON) biosynthesis functions [16]. In *M. oryzae*, two transcript isoforms of MoPTEN, produced by alternative splicing, are involved in spore germination, hyphal growth, and pathogenicity [17]. To date, there have been no reports for identification of alternative splicing events in *B. cinerea* during the infection stage.

Previously, we released the high-quality RNA-sequencing data at different infection stages including conidia germination (6 h), pre-penetration (12 h), biotrophic stage (24 h), and necrotrophic stage (48 h). In this study, these datasets were analyzed to determine the type, frequency, and infection-stage-specific patterns of genes and mRNA isoforms affected by alternative splicing in *B. cinerea*. The concept of isoform abundance was used to assess whether transcriptional function changes under different conditions.

## 2. Materials and Methods

### 2.1. Transcriptome Analysis

We downloaded published transcriptome datasets generated from NCBI (Accession No. PRJNA1056687). RNA-Seq data were aligned to the *B. cinerea* genome (ASM83294v1, Ensembl fungi v53) using HISAT2 v2.04 [18]. De novo annotation was assembled using StringTie v2.1.3. [19]. Expression values (Transcripts per kilobase million, TPM) of transcripts were calculated using Salmon v1.9 [20].

### 2.2. Identification of Alternative Splicing

Alternative splicing landscapes were obtained by using SUPPA v2.2.1. The percent splicing index (PSI) for each alternative splicing event was calculated using SUPPA2 v2.2.1 [21]. Salmon v1.9 [20] was employed to calculate the transcripts per million (TPM) of transcripts in each sample. The DESeq2 v1.24.0 [22] tool was utilized to assess fold changes in transcript expression values. Transcripts were deemed differentially expressed based on comparisons between treatment and control groups.

### 2.3. Gene Function Analysis

Transcripts that contained any recognized Pfam domains and non-coding RNAs were excluded based on the InterProScan v68 [23] and Rfam v14.1 [24] databases. The coding potential of the transcripts was assessed using CPAT v1.2.2 [25]. All proteins that might contain signal peptides were evaluated for their potential for secretion using SignalP 5.0b [26] and TMHMM-2.0c [27].

### 2.4. RNA Extraction and RT-PCR Validation

The culture of plant and pathogenic fungi and the assays of pathogen infection were performed as described previously [28]. The hyphae samples were collected after 6 h, 12 h, 24 h, and 48 h of infection stages, respectively. Normal growth hyphae at corresponding time points were also collected as controls. Total RNA was isolated from *B. cinerea* using a total RNA extraction kit (RNAsimple Total RNA Kit; Tiangen Biotech, Beijing, China). First-strand complementary DNA (cDNA) was synthesized using the TransScript^®^ One-Step gDNA Removal and cDNA Synthesis SuperMix (Transgen, Beijing, China). RT-PCR was performed using gene-specific primers (Table S2), and the procedures included an initial 95 °C denaturation step for 3 min, followed by denaturation for 15 s at 95 °C, annealing for 15 s at 55 °C, and extension for 1 min at 72 °C for 30 cycles.

## 3. Results

### 3.1. Spliceosome Components Upregulate Expression During Infection in B. cinerea

**Figure 1:**
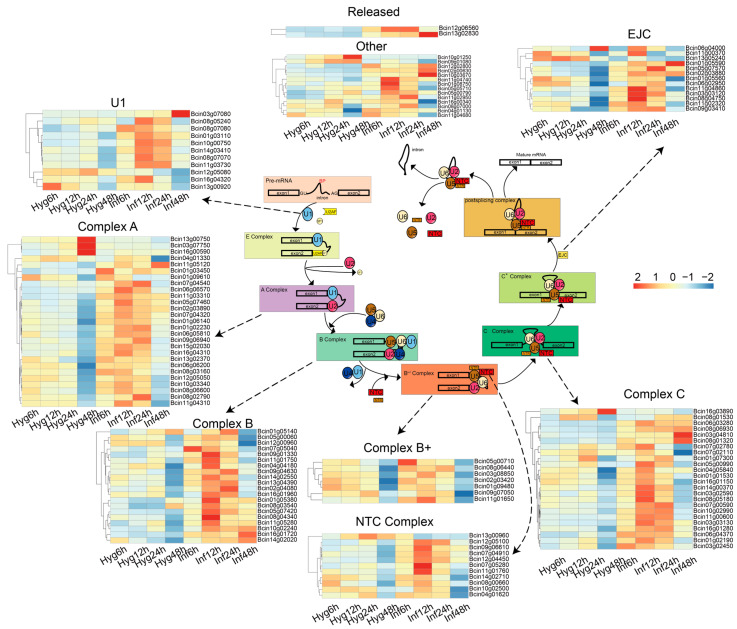
Identification of spliceosome components and their transcriptional regulation in *B. cinerea* during the infection across four consecutive stages. The schematic diagram illustrates the mRNA splicing process in *B. cinerea* genes. A pre-mRNA is displayed as a black stroke box with two exons, and an intron is displayed as a black line. Circular and square shapes symbolize protein complexes. The outer section features a heat map indicating the expression changes of 129 spliceosome-related genes in *B. cinerea* across the hyphal and infection hyphal stages. High expression levels (orange to red) and low expression levels (yellow to blue) are represented as z-scores for each gene. Pre-mRNA splicing involves several spliceosomal complexes, including Complex E, which is formed by the binding of U1 snRNP to small nuclear ribonucleoprotein-related proteins; U2-related factor (U2AF) and the bonding factor (SF) come together to create complex A; U4/U5/U6 tri-snRNP spliceosomal complex is stabilized by the PRP19C/Prp19 complex/NTC/19 complex, facilitating the assembly of complex B. Release of U1 and U4 snRNPs leads to the formation of the activated spliceosome complex B+, which triggers branching, intron excision, and conformational rearrangement into complex C. * represents a state of catalytic activation.

RNA splicing is a critical step in the generation of mature mRNA, a process accomplished by the spliceosome. In order to study alternative splicing in *B. cinerea*, we first identified the homologous genes of splicing-related factors through BLASTp v2.5.0. A total of 129 genes about all the main components across pre-mRNA splicing cycle were identified, including U1/U2/U4/U5/U6-associated components, NineTeen Complex (NTC), exon junction complex (EJC), the mRNA and intron release components (Figure 1; Table S1).

We utilized RNA-seq reads of *B. cinerea* cultured in vitro on potato dextrose broth (PDB) to examine the transcriptional regulation of the spliceosome components during four consecutive infection stages (Figure 1). We found that 129 spliceosome-related genes expressed more than 10 TPM (Transcripts per kilobase million) in at least one stage, except Bcin01p03450 (Complex A, encoding U2-related factor). Surprisingly, many spliceosome components exhibited high expression during the infection, especially at key nodes (12 h and 24 h) where the fungus infects the host (Figure 1). Among the 129 genes, 104 genes were upregulated in at least one infection stage, and 44 genes (*p*-value < 0.05 and log_2_ fold change ≥1) were differentially upregulated. For instance, Bcin10g03670 demonstrated a remarkable increase in expression, rising 113-fold at 48 h during the infection stage compared to the vegetative stage (Table S3). These results suggested that the spliceosome components are specifically highly expressed during the infection stage.

### 3.2. Most of Alternative Splicing Is Stage-Specific in B. cinerea

**Figure 2:**
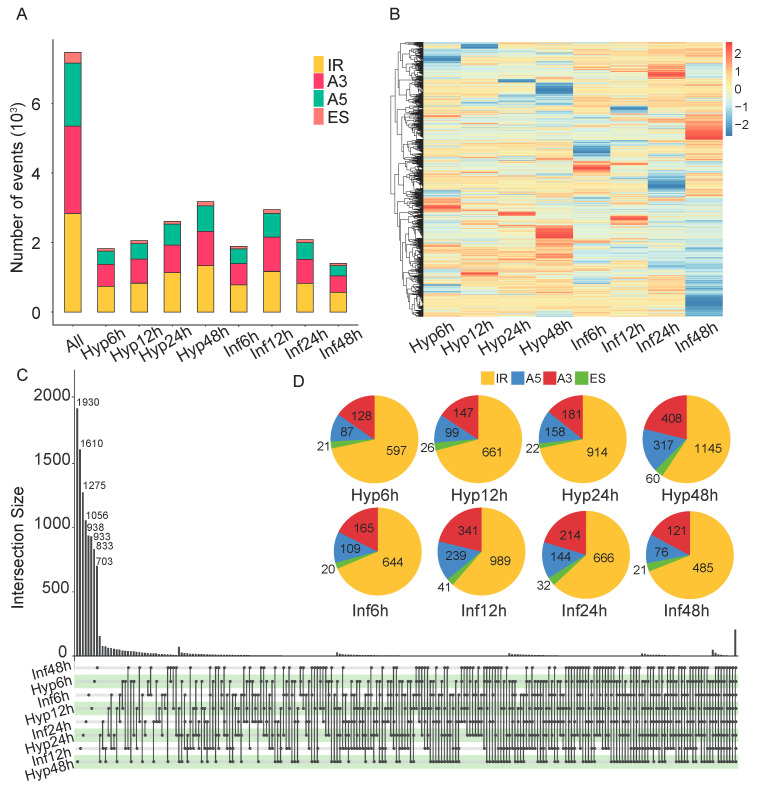
Identification and expression of infection-associated alternating splicing events in *B. cinerea.* (**A**) Statistics of the number of alternative splicing events and types. IR: intron retention. A5: alternative 5′-donor. A3: alternative 3′-acceptor. ES: exon skip. (**B**) Heatmap of Percent Spliced-In (PSI) values during eight stages. High expression levels (orange to red) and low expression levels (yellow to blue) are represented as z-scores for each alternative splicing event. (**C**) Overlap of alternative splicing events across different stages. (**D**) Statistics of tissue-specific alternative splicing types in different stages.

Across all samples, we identified a total of 7466 alternative splicing events in 3308 genes (Figure 2A). There are four major types of alternative splicing, including intron retention (IR), alternative 5′-donor (A5), alternative 3′-acceptor (A3) and exon skip (ES). The most abundant alternative splicing event is IR, followed by A5 and A3, while ES is the least common (Figure 2A). The percent splicing index (PSI) values of alternative splicing events were higher in Hyp48h but lower in Inf24h and Inf48h (Figure 2B). Surprisingly, tissue of Hyp48h has the highest number of specific alternative splicing events, whereas Inf48h has the least amount of specific splicing events (Figure 2C). Importantly, IR is the most frequent specific alternative splicing type among all stages (Figure 2D). Taken together, these results suggest that alternative splicing is extremely stage-specific, especially during the host infection stage in *B. cinerea*.

### 3.3. Validation of Alternative Splicing Events in B. cinerea

**Figure 3:**
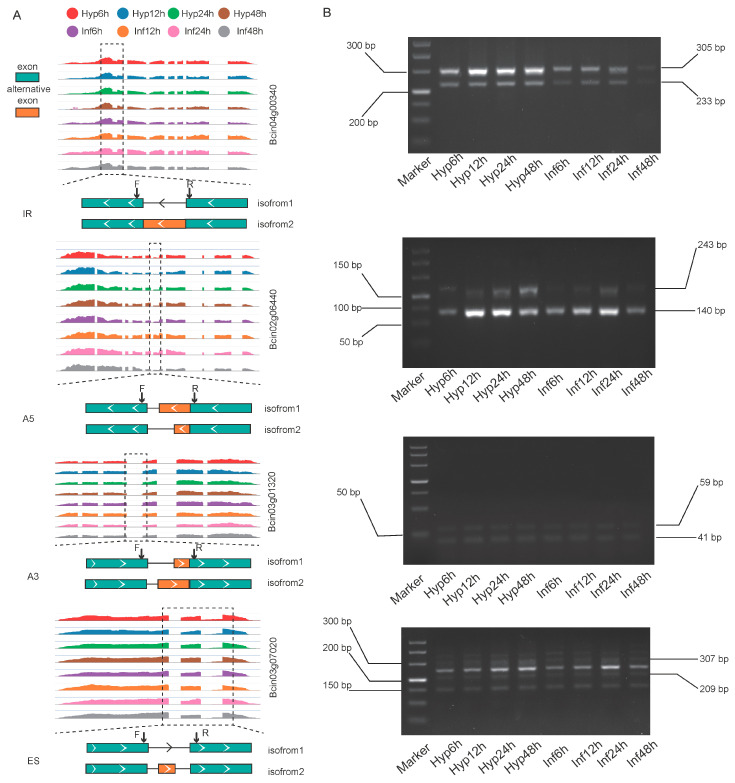
Validation of four types of alternative splicing events across eight stages using RT-PCR. (**A**) The RNA-Seq read coverage of the four types of alternative splicing events (IR: intron retention, A5: alternative 5′-donor, A3: alternative 3′-acceptor, ES: exon skip) across eight developmental stages (hyphae: Hyp6h, Hyp12h, Hyp24h, Hyp48h; infection hyphae: Inf6h, Inf12h, Inf24h, Inf48h) is presented. Eight distinct colors represent the different stages. Below each alternative splicing event, the corresponding transcript is displayed, with green indicating constitutive exons and orange denoting alternative exons. PCR primers (F for forward and R for reverse) are designed to flank the alternative exons. (**B**) RT-PCR validation was conducted on the four types of alternative splicing (AS) events at the marked loci, utilizing RNA isolated from hyphae and infection hyphae. The left side of the gel displays the sizes of the DNA markers, while the right side presents the gel bands. The dashed line box highlights the regions where alternative splicing occurs.

Four genes were randomly selected to validate the accuracy of alternative splicing (AS) events using reverse transcription polymerase chain reaction (RT-PCR). To design primers that could amplify all expected transcripts simultaneously, the isoforms of each gene were aligned (Figure 3A; Table S2). The results indicated that the size of each amplified fragment, as demonstrated by the gel banding pattern, corresponded with the predicted fragment sizes (Figure 3B).

### 3.4. Analysis of Coding Capabilities of Transcripts Generated by Alternative Splicing in B. cinerea

**Figure 4:**
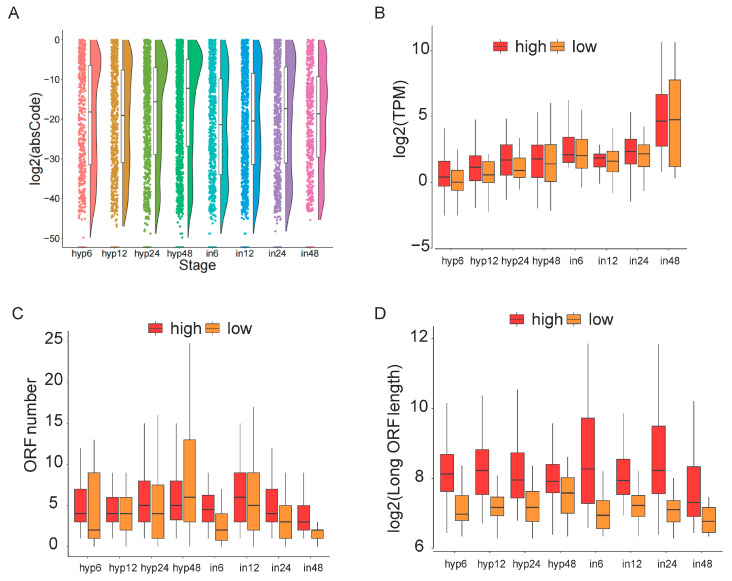
Changes in isoform coding potential resulting from alternative splicing. (**A**) Absolute value of change in coding potential of transcripts generated by alternative splicing. (**B**–**D**) Comparison of expression values, open reading frame (ORF) numbers and ORF length of transcripts with high coding potential and transcripts with low coding potential generated by alternative splicing.

The pre-mRNAs can also undergo alternative splicing to generate more than one protein-coding mRNAs from a gene. To explore changes in the coding capacity of transcripts after alternative splicing, the multiple coding potential indicators of transcripts were analyzed. Firstly, we compared the coding ability of the two isoforms generated by alternative splicing from one gene. In the hyphal stage, the coding capacity among transcripts produced by alternative splicing was generally greater than in the infection stage (Figure 4A). The expression levels of transcripts with high coding potential were generally higher than those of transcripts with low coding potential in all stages (Figure 4B). The following analysis results also showed that transcripts with high coding potential had higher number and length of open reading frames (ORFs) than transcripts with low coding potential (Figure 4C,D).

**Figure 5:**
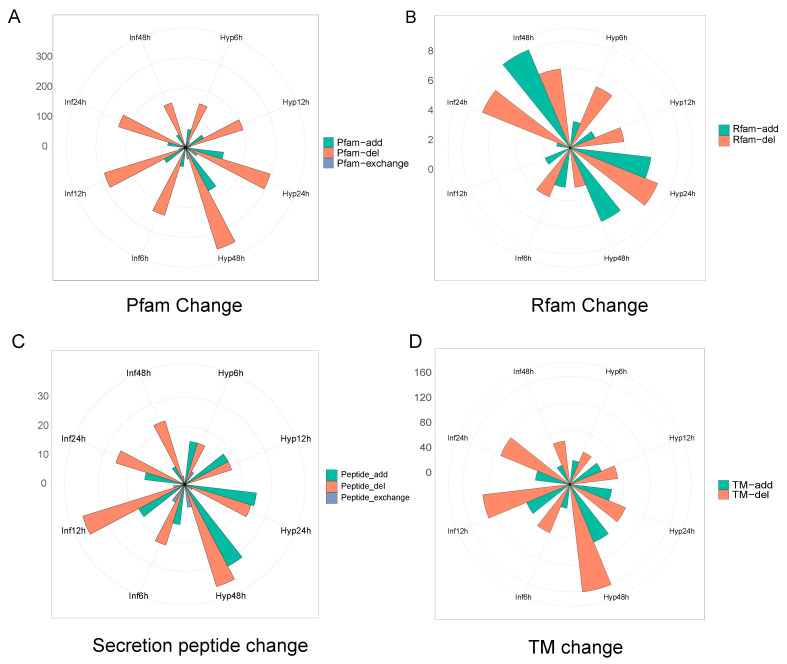
Changes in isoform functional domain resulting from alternative splicing. (**A**–**D**) Changes in protein family, RNA family, secretion peptide and transmembrane helices (THMM) between transcripts generated by alternative splicing.

Furthermore, we explored the changes in functional domains between transcripts generated by alternative splicing. Using high coding potential transcripts as controls, we compared isoform structural changes resulting from alternative splicing, which were divided into three types: domain increase, domain decrease, and domain exchange. Compared with transcripts with high coding potential, transcripts with low coding potential produced by alternative splicing at all stages have mostly lost protein domains (Figure 5A). Compared with changing the number of protein families (Pfam), alternative splicing affects the number of RNA families (Rfam) much less (Figure 4B). Except for the Hyp48 h, Rfam reduction was the predominant type among transcripts with low coding potential (Figure 4B).

In the study of host infection by plant pathogenic fungi, secreted proteins represent a critical area of research. To investigate whether alternative splicing influences the function of effector proteins, we assessed the effects of alternative splicing on the signal peptide and transmembrane domains. Throughout the hyphal stages, alternative splicing produces transcript isoforms that consistently increase the number of signal peptides while simultaneously decreasing the overall count of signal peptides. During the infection stage, transcripts with reduced signal peptides were significantly more abundant than those with increased signal peptides (Figure 4C). However, reduction in the transmembrane domains of transcripts encoding low potential is the predominant type (Figure 4D). These results indicate that transcripts generated by alternative splicing with high coding potential exhibit higher expression levels and contain more functional domains.

## 4. Discussion

Alternative splicing represents a crucial mechanism of gene regulation in eukaryotic cells. It is not uncommon for a single gene to yield proteins with distinct functions [29]. In humans, the RNA-binding protein PTBP1 controls Sertoli cell actin cytoskeletal reorganization by promoting actin bundle formation by inhibiting exon 14 inclusion of Tnik [30]. In wheat, the *TaNAK1* gene is present in two isoforms. TaNAK1.1 features an exon deletion that leads to a truncated protein kinase domain in the encoded protein. The *TaNAK1* gene plays a crucial role in regulating plant height, branch number, seed size, and total yield by enhancing the expression of TaNAK1.1 or TaNAK1.2 isoforms [31]. However, this type of exploration of large-scale changes in gene coding regions has not been extensively conducted in fungi, particularly in *B. cinerea*. In this study, we characterized the alternative splicing events that occur in *B. cinerea* during the host-infection stage. Furthermore, we experimentally validated the accuracy of the predicted alternative splicing results. Additionally, we conducted a preliminary exploration of the encoding potential of transcript isoforms generated by alternative splicing.

Although the majority of spliceosome components are expressed universally during development, there are particular expression tendencies for certain types of tissues and specific stages of development. In our analysis, we found that many spliceosome components are upregulated in expression during infection in *B. cinerea*. Notably, we found that most spliceosome genes exhibit high expression levels at critical time points during the infection process, including the pre-infection stage of germinating spores (12 h) and the stage when nascent hyphae invade the interior of the host (24 h) in *B. cinerea* [32]. This upregulated expression of spliceosome genes during the infection stage may vary according to the infection strategies of different plant pathogenic fungi. Contrary to the results in *B. cinerea*, most spliceosome genes were downregulated during the *S. sclerotiorum* infection stage, which may be affected by host defense [14,15]. We speculate that the phenomenon that spliceosome genes are generally upregulated during the infection stage may be related to the anti-host immune mechanism of *B. cinerea*.

The identification of alternative splicing events has commenced in plant pathogenic fungi. In *F. graminearum*, approximately 5000 genes are involved in alternative splicing [9]. A total of 1487 genes exhibited differentially expressed alternative splicing events in various hosts infected by *S. sclerotiorum* [15]. In this study, alternative splicing was found to be widespread in *B. cinerea*. Furthermore, during the host infection stage, a significant number of stage-specific alternative splicing events are generated. A total of 3308 genes were alternatively spliced and generated 7466 alternative splicing events in *B. cinerea*. Only 205 of these alternative splicing events were expressed across all stages, with the majority being stage-specific. This may be related to the tissue growth, development and pathogenicity requirements of fungi at different growth and infection stages.

Alternative splicing expands the coding capacity of eukaryotic genomes, allowing a limited number of genes to control the development of complex structures [33]. In this study, we conducted a statistical analysis on the changes in the coding capacity of transcripts produced by alternative splicing. The results indicate that the coding ability associated with alternative splicing varies significantly during the hyphal stage, while changes are minimal during the infection stage. The generation of alternative splicing is significantly influenced by external conditions and environmental stressors. As the impact of environmental stress intensifies, conserved intronic sites are preferentially selected for splicing. Conversely, when environmental pressure is low, novel intronic sites are chosen for splicing, resulting in the production of new transcripts and enhanced protein diversity, thereby facilitating adaptation to evolutionary changes [34]. This discrepancy may be attributed to the mycelial stage experiencing less survival pressure, which increases the likelihood of incorrect splicing, whereas the rate of incorrect splicing is reduced during the infection stage. In this study, alternative splicing produces transcripts that exhibit reductions in various functional domains compared to the predominantly expressed transcript, including Pfam domains, signal peptides for secretion, and transmembrane domains. We conclude that the majority of alternative splicing events are erroneous and lack functional significance. However, we found that alternative splicing affecting protein functional domains is diminished during the infection stage. But it cannot be completely denied that there are functional examples of these alternative splicing events. For example, the CPEB (cytoplasmic polyadenylation element binding family) gene balances the development of idiopathic autism spectrum disorder through transcript isoforms generated by mis-splicing.

## 5. Conclusions

Overall, our study presents the first identification of alternative splicing events in *B. cinerea*. The results confirm that these alternative splicing events are highly stage-specific. Notably, the coding capacity associated with alternative splicing varies significantly during the hyphal stage, whereas changes during the infection stage are minimal. While most alternative splicing events appear to be erroneous and lack functional significance, we observed that the impact of alternative splicing on protein functional domains diminishes during the infection stage. Collectively, our findings offer new insights into the underlying mechanisms through which *B. cinerea* infects its hosts.

## Data Availability

Data are contained within the article (https://www.ncbi.nlm.nih.gov/search/all/?term=PRJNA1056687 accessed on 17 January 2025).

## Supplementary Materials

The following supporting information can be downloaded at: https://www.mdpi.com/article/10.3390/microorganisms13020360/s1, Table S1: List of spliceosome-related genes in *B. cinerea*, Table S2: The primer pairs used in this study, Table S3: Different expression of 129 splicing complex-related genes in *B. cinerea*.

## References

[1] Wang F., Jin Z.X., Wang S.N., Yang L.C., Fan Z.B., Yao Y.X. (2024). ASAPA: A bioinformatic pipeline based on Iso-Seq that identifies the links among alternative splicing, alternative transcription initiation and alternative polyadenylation. Funct. Integr. Genom..

[2] Gallego-Paez L.M., Bordone M.C., Leote A.C., Saraiva-Agostinho N., Ascensao-Ferreira M., Barbosa-Morais N.L. (2017). Alternative splicing: The pledge, the turn, and the prestige:The key role of alternative splicing in human biological systems. Hum. Genet..

[3] Baralle F.E., Giudice J. (2017). Alternative splicing as a regulator of development and tissue identity. Nat. Rev. Mol. Cell Biol..

[4] Bae B., Miura P. (2020). Emerging Roles for 3′ UTRs in Neurons. Int. J. Mol. Sci..

[5] Carrasco J., Rauer M., Hummel B., Grzejda D., Alfonso-Gonzalez C., Lee Y., Wang Q.Q., Puchalska M., Mittler G., Hilgers V. (2020). ELAV and FNE Determine Neuronal Transcript Signatures through EXon-Activated Rescue. Mol. Cell.

[6] Liu R., Zhao D., Li P., Xia D., Feng Q., Wang L., Wang Y., Shi H., Zhou Y., Chen F. (2024). Natural variation in *OsMADS1* transcript splicing affects rice grain thickness and quality through influencing monosaccharide loading to endosperm. Plant Commun..

[7] Kawashima T., Douglass S., Gabunilas J., Pellegrini M., Chanfreau G.F. (2014). Widespread Use of Non-productive Alternative Splice Sites in *Saccharomyces cerevisiae*. PLoS Genet..

[8] Schirman D., Yakhini Z., Pilpel Y., Dahan O. (2021). A broad analysis of splicing regulation in yeast using a large library of synthetic introns. PLoS Genet..

[9] Lu P., Chen D.P., Qi Z.M., Wang H.M., Chen Y.T., Wang Q.H., Jiang C., Xu J.R., Liu H.Q. (2022). Landscape and regulation of alternative splicing and alternative polyadenylation in a plant pathogenic fungus. New Phytol..

[10] Jeon J., Kim K.T., Choi J., Cheong K., Ko J., Choi G., Lee H., Lee G.W., Park S.Y., Kim S. (2022). Alternative splicing diversifies the transcriptome and proteome of the rice blast fungus during host infection. RNA Biol..

[11] Jin L.R., Li G.L., Yu D.Z., Huang W., Cheng C., Liao S.J., Wu Q.J., Zhang Y. (2017). Transcriptome analysis reveals the complexity of alternative splicing regulation in the fungus *Verticillium dahliae*. BMC Genom..

[12] Veloso J., van Kan J.A.L. (2018). Many Shades of Grey in *Botrytis*-Host Plant Interactions. Trends Plant Sci..

[13] Spada M., Pugliesi C., Fambrini M., Pecchia S. (2024). Challenges and Opportunities Arising from Host- *Botrytis cinerea* Interactions to Outline Novel and Sustainable Control Strategies: The Key Role of RNA Interference. Int. J. Mol. Sci..

[14] Cheng X.H., Zhao C.J., Gao L.X., Zeng L.Y., Xu Y., Liu F., Huang J.Y., Liu L.J., Liu S.Y., Zhang X. (2022). Alternative splicing reprogramming in fungal pathogen *Sclerotinia sclerotiorum* at different infection stages on *Brassica napus*. Front. Plant Sci..

[15] Ibrahim H.M.M., Kusch S., Didelon M., Raffaele S. (2021). Genome-wide alternative splicing profiling in the fungal plant pathogen *Sclerotinia sclerotiorum* during the colonization of diverse host families. Mol. Plant Pathol..

[16] Zhang Y.M., Gao X.L., Sun M.L., Liu H.Q., Xu J.R. (2017). The *FgSRP1* SR-protein gene is important for plant infection and pre-mRNA processing in *Fusarium graminearum*. Environ. Microbiol..

[17] Wang S.W., Liang H., Wei Y., Zhang P.H., Dang Y.J., Li G.H., Zhang S.H. (2021). Alternative Splicing of Is Important for Growth and Pathogenesis in *Magnaporthe oryzae*. Front. Microbiol..

[18] Kim D., Paggi J.M., Park C., Bennett C., Salzberg S.L. (2019). Graph-based genome alignment and genotyping with HISAT2 and HISAT-genotype. Nat. Biotechnol..

[19] Pertea M., Kim D., Pertea G.M., Leek J.T., Salzberg S.L. (2016). Transcript-level expression analysis of RNA-seq experiments with HISAT, StringTie and Ballgown. Nat. Protoc..

[20] Patro R., Duggal G., Love M.I., Irizarry R.A., Kingsford C. (2017). Salmon provides fast and bias-aware quantification of transcript expression. Nat. Methods.

[21] Trincado J.L., Entizne J.C., Hysenaj G., Singh B., Skalic M., Elliott D.J., Eyras E. (2018). SUPPA2: Fast, accurate, and uncertainty-aware differential splicing analysis across multiple conditions. Genome Biol..

[22] Love M.I., Huber W., Anders S. (2014). Moderated estimation of fold change and dispersion for RNA-seq data with DESeq2. Genome Biol..

[23] Mitchell A.L., Attwood T.K., Babbitt P.C., Blum M., Bork P., Bridge A., Brown S.D., Chang H.Y., El-Gebali S., Fraser M.I. (2019). InterPro in 2019: Improving coverage, classification and access to protein sequence annotations. Nucleic Acids Res..

[24] Ontiveros-Palacios N., Cooke E., Nawrocki E.P., Triebel S., Marz M., Rivas E., Griffiths-Jones S., Petrov A., Bateman A., Sweeney B. (2024). Rfam 15: RNA families database in 2025. Nucleic Acids Res..

[25] Wang L., Park H.J., Dasari S., Wang S., Kocher J.P., Li W. (2013). CPAT: Coding-Potential Assessment Tool using an alignment-free logistic regression model. Nucleic Acids Res..

[26] Armenteros J.J.A., Tsirigos K.D., Sonderby C.K., Petersen T.N., Winther O., Brunak S., von Heijne G., Nielsen H. (2019). SignalP 5.0 improves signal peptide predictions using deep neural networks. Nat. Biotechnol..

[27] Möller S., Croning M.D., Apweiler R. (2001). Evaluation of methods for the prediction of membrane spanning regions. Bioinformatics.

[28] Shi H.J., Ding G.J., Wang Y., Wang J.Q., Wang X.L., Wang D., Lu P. (2025). Genome-wide identification of long non-coding RNA for *Botrytis cinerea* during infection to tomato (*Solanum lycopersicum*) leaves. BMC Genom..

[29] Jiang J., Wu H., Ji Y., Han K., Tang J.M., Hu S., Lei W. (2024). Development and disease-specific regulation of RNA splicing in cardiovascular system. Front. Cell Dev. Biol..

[30] Wang Y.X., Chembazhi U.V., Yee D., Chen S.J., Ji J., Wang Y.J., Nguyen K.L., Lin P.C., Ratti A., Hess R.A. (2024). PTBP1 mediates Sertoli cell actin cytoskeleton organization by regulating alternative splicing of actin regulators. Nucleic Acids Res..

[31] Wu B.W., Zhang X.Y., Hu K.Z., Zheng H.Y., Zhang S.Y., Liu X.L., Ma M., Zhao H.X. (2022). Two alternative splicing variants of a wheat gene *TaNAK1*, *TaNAK1.1* and *TaNAK1.2*, differentially regulate flowering time and plant architecture leading to differences in seed yield of transgenic *Arabidopsis*. Front. Plant Sci..

[32] Bi K., Liang Y., Mengiste T., Sharon A. (2023). Killing softly: A roadmap of *Botrytis cinerea* pathogenicity. Trends Plant Sci..

[33] Murphy D., Cieply B., Carstens R., Ramamurthy V., Stoilov P. (2016). The Musashi 1 Controls the Splicing of Photoreceptor-Specific Exons in the Vertebrate Retina. PLoS Genet..

[34] Xing Y., Lee C. (2006). Alternative splicing and RNA selection pressure--evolutionary consequences for eukaryotic genomes. Nat. Rev. Genet..

